# Choroidal Microvascular Dropout in Pseudoexfoliation Glaucoma

**DOI:** 10.1167/iovs.19-26844

**Published:** 2019-05

**Authors:** Zia S. Pradhan, Harsha L. Rao, Shivani Dixit, Shruthi Sreenivasaiah, Praveena G. Reddy, Jayasree P. Venugopal, Narendra K. Puttaiah, Sathi Devi, Robert N. Weinreb, Kaweh Mansouri, Carroll A. B. Webers

**Affiliations:** 1 Narayana Nethralaya, Rajajinagar, Bangalore, India; 2 Narayana Nethralaya, Hulimavu, Bangalore, India; 3 Shiley Eye Institute, Hamilton Glaucoma Center and Department of Ophthalmology, University of California, San Diego, California, United States; 4 Glaucoma Center, Montchoisi Clinic, Swiss Vision Network, Lausanne, Switzerland; 5 Department of Ophthalmology, University of Colorado, Denver, Colorado, United States; 6 University Eye Clinic Maastricht, University Medical Center, Maastricht, the Netherlands

**Keywords:** choroidal microvascular dropout, optical coherence tomography angiography, pseudoexfoliation glaucoma, primary open angle glaucoma

## Abstract

**Purpose:**

To compare the prevalence of choroidal microvasculature dropout (CMvD) in pseudoexfoliation glaucoma (PXG) and disease severity–matched primary open-angle glaucoma (POAG) eyes.

**Methods:**

In a cross-sectional study, 39 eyes with PXG (33 patients) and 39 glaucoma severity–matched POAG eyes (34 patients) underwent visual fields, optical coherence tomography and optical coherence tomography angiography examination. Peripapillary vessel density (VD) was evaluated from the radial peripapillary capillary slab, parafoveal VD was measured on the superficial vascular plexus slab of the macula, and CMvD was evaluated on the choroidal slabs of the optic disc scan.

**Results:**

The PXG and POAG groups were similar with respect to average mean deviation on visual fields (−12.1 vs. −12.0 decibel, *P* = 0.96) and average peripapillary retinal nerve fiber layer thickness on optical coherence tomography (71 vs. 74 μ, *P* = 0.29). Average peripapillary superficial VD (49.7% vs. 51.3%, *P* = 0.35) and parafoveal VD (44.8% vs. 45.8%, *P* = 0.33) were similar between the PXG and POAG groups. CMvD was seen in 18 PXG and 31 POAG eyes (46.2% vs. 79.5%, *P* = 0.002). On multivariate analysis that accounted for the severity of glaucoma, the odds of CMvD was significantly lower in the PXG group when compared with the POAG group (odds ratio: 0.18–0.21, *P* < 0.01).

**Conclusions:**

The prevalence of CMvD was significantly lower in the PXG eyes when compared with the POAG eyes.

Pseudoexfoliation glaucoma (PXG) is a type of glaucoma that is characterized by the presence of a fibrillar, extracellular material on various tissues in the anterior segment of the eye. The deposition of this material causes trabecular dysfunction, resulting in high intraocular pressure (IOP) and optic nerve damage. When compared with primary open angle glaucoma (POAG), PXG eyes have a higher IOP and a faster rate of glaucomatous progression.^1^ PXG is also associated with a vasculopathy, which is characterized by abnormalities of the endothelial basement membrane and obliteration of the vascular lumen.^2^ Vascular alterations involving PXG eyes include iris vessel dropout and collateral formation, which has been studied using fluorescein angiography and histopathology, retinal vein occlusions determined on slit-lamp biomicroscopy, and impaired retrobulbar blood flow reported using color Doppler imaging.^1^–^3^

Recently, optical coherence tomography angiography (OCTA) has been used to noninvasively quantify the superficial blood vessels of the optic nerve head (ONH) and peripapillary and macular regions as well as the deeper choroidal vasculature of glaucoma patients.^4^ Reduction in the superficial retinal vessel density of the peripapillary and the macular regions in POAG eyes has been demonstrated using OCTA.^5^–^11^ In addition, the complete loss of choriocapillaris in localized regions of parapapillary atrophy (PPA), called choroidal microvasculature dropout (CMvD), has been observed using OCTA in POAG eyes.^12^–^14^

Studies evaluating the OCTA changes in PXG are limited. A few recent studies have evaluated the superficial retinal vasculature in the peripapillary region of PXG eyes and have reported that the reduction of vessel densities was greater in PXG eyes when compared with POAG eyes of similar disease severity.^15^,^16^ However, there have been no reports of the prevalence of CMvD in PXG. The purpose of the current study was to compare the prevalence of CMvD in PXG and disease severity–matched POAG eyes.

## Methods

This was a prospective, cross-sectional study conducted at Narayana Nethralaya, a tertiary eye care center in Bengaluru, South India, between September 2015 and September 2017. The methodology adhered to the tenets of the Declaration of Helsinki for research involving human subjects. Written informed consent was obtained from all participants, and the study was approved by the institute's ethics committee.

Participants of the study included PXG and POAG patients. All patients had open angles on gonioscopy and glaucomatous changes of the ONH as documented by glaucoma experts on dilated fundus examination and confirmed by experts on stereoscopic optic disc photographs. Glaucomatous changes on the ONH included neuroretinal rim thinning, notching, and retinal nerve fiber layer defects. All patients also had visual field (VF) defects correlating with the ONH findings. In addition to these findings, the PXG patients had pseudoexfoliation (PXF) deposits on the pupillary margin and anterior lens capsule, visible after mydriasis on slit-lamp biomicroscopy. Inclusion criteria for all participants were age ≥18 years, corrected distance visual acuity of 20/40 or better, and refractive error within ±5 diopter (D) sphere and ±3 D cylinder. Exclusion criteria were the presence of any media opacities that prevented good-quality optical coherence tomography (OCT) scans or any retinal or neurological disease other than glaucoma that could confound the evaluation.

All consecutive PXG and POAG patients attending the glaucoma clinic between September 2015 and September 2017 were recruited. All participants underwent a comprehensive ocular examination along with stereoscopic optic disc photography, VF examination, and OCT imaging with RTVue-XR spectral-domain OCT (SD-OCT) (Optovue Inc., Fremont, CA, USA).

Stereoscopic optic disc photographs were evaluated independently by two glaucoma experts in a masked manner to determine the presence of glaucomatous changes as described previously.^11^,^14^

VF examination was performed using a Humphrey Field analyzer II, model 720i (Zeiss Humphrey Systems, Dublin, CA, USA), with the Swedish interactive threshold algorithm standard 24-2 program. VF was considered glaucomatous if the pattern standard deviation was abnormal with a probability value *P* < 5%, a glaucoma hemifield test was outside normal limits, and the pattern deviation probability plot contained ≥3 points in a cluster depressed to a *P* < 5% level with one or more of these points depressed to a *P* < 1% level. The type of VF defect was also determined based on a previously described classification.^17^ An initial parafoveal scotoma was defined as a glaucomatous VF defect within 12 points of the central 10° of fixation, and no VF defect in the nasal periphery outside 10° of fixation. An initial nasal defect was defined as a glaucomatous VF defect in the nasal periphery outside the central 10° of fixation, with no VF defect within the central 10°. A combined defect (nasal defect and parafoveal scotoma) was defined as a glaucomatous defect involving both the nasal and parafoveal regions.

OCTA imaging of the optic disc and the macular region was performed using RTVue-XR SD-OCT (version 2016.1.0.26; Optovue Inc.) as detailed previously.^11^ The optic disc OCTA scan is performed using volumetric scans covering an area of 4.5 × 4.5 mm, and the software automatically fits an ellipse to the optic disc margin. The peripapillary region is defined as a 0.75-mm wide elliptical annulus extending from the optic disc boundary. This region is divided into six sectors based on the Garway-Heath map.^18^ The vessel density in the entire peripapillary regions and in each of these sectors was calculated from the radial peripapillary capillary segment, which extends from the internal limiting membrane to the posterior boundary of the retinal nerve fiber layer (RNFL).

The macular OCTA scan was performed using volumetric scans covering a 3 × 3 mm area. The macular vessel densities were analyzed within a 1.5-mm wide parafoveal, circular annulus centered on the fovea. This region was divided in two hemispheres of 180° each (superior and inferior) for the calculation of vessel densities. In addition, it was also divided into four sectors of 90° each (nasal, inferior, superior, and temporal sectors), and the vessel densities in each sector were reported. The macular vessel densities analyzed were of the superficial vascular plexus present between the internal limiting membrane and the inner plexiform layer.

### Evaluation of the Choroidal OCTA Slab

The parapapillary CMvD was evaluated on the enface images of the choroidal slabs segmented automatically by the OCTA software (Figure, panel a). CMvD was defined as a focal, sectoral capillary dropout (no visible microvasculature) within the area of PPA, the circumferential width of which was more than one half clock hour of the disc circumference.^19^

**Figure:**
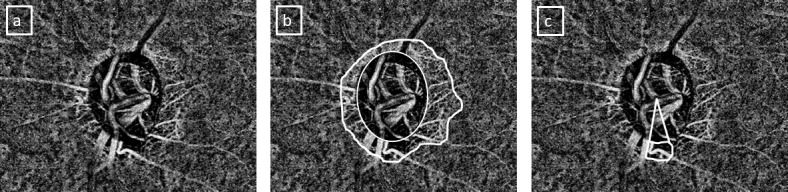
Evaluation of choroidal microvascular dropout (CMvD) in an eye with pseudoexfoliation glaucoma. (a) Choroidal slab of an optical coherence tomography angiography scan. (b) Optic disc margin (inner circle) and the region of parapapillary atrophy (outer outline) marked on the choroidal slab. (c) CMvD and the angular extent of CMvD marked on the slab.

The following measurements were obtained from the choroidal OCTA slab using ImageJ software (version 1.51; National Institutes of Health, Bethesda, MD, USA) as described previously:^14^ optic disc area, area of PPA, CMvD area, and angular extent of CMvD. Optic disc and the PPA margins were marked by simultaneously viewing the stereoscopic optic disc photographs and the scanning laser ophthalmoscopic images that were obtained along with the OCTA images (Figure, panel b). The borders of the CMvD were manually delineated, and the area and angular extent were measured using the automated software (Figure, panel c). In an eye showing more than one CMvD, the area and the angular extent of each CMvD was added to determine the total area and total angular extent of CMvD for the eye. Poor-quality choroidal slab images, defined as those with blurred images that hampered the delineation of the disc margins, PPA, or CMvD, were excluded from the analysis. The identification of CMvD and all of the aforementioned measurements were done independently by two examiners (S.S. and S.D.) who were masked to the clinical, VF, and RNFL details of the patients. Disagreements between the observers were resolved by a third adjudicator (H.L.R.).

All of the participants also underwent the peripapillary RNFL thickness and macular ganglion cell complex (GCC) measurements on the RTVue-XR SD-OCT using the ONH and GCC scans, respectively. These scan protocols were previously explained in detail.^20^,^21^ All of the examinations for a particular participant were performed on the same day.

After the recruited participants underwent all of the aforementioned tests, the VFs and OCT and OCTA scans were reviewed for quality. VFs were considered reliable if fixation losses were less than 20% and false positives and false negative response rates were less than 15%. Unreliable VFs were excluded. Poor-quality OCT and OCTA scans, defined as those with a signal strength index <35, or scans with motion artifacts and segmentation errors were also excluded from the analysis. Following these exclusions, once the study cohort of PXG eyes was finalized, severity-matched POAG eyes were selected from the recruited participants. Mean deviation (MD) on VF was the criterion used to match individuals of the two cohorts.

### Statistical Analysis

The sample size required for the determination of a difference of 30% in the prevalence of CMvD between the two groups at a power of 80%, and a two-sided α level of 5% was calculated to be 39 in each group.

The descriptive statistics included the mean and standard deviation for continuous variables and the percentages for categorical variables. Interexaminer agreement regarding the presence of CMvD and the measurements from the choroidal OCTA slab were assessed using κ statistics and intraclass correlation coefficients. Factors associated with the presence of CMvD in the entire cohort of open angle glaucoma eyes were initially evaluated using univariate logistic regression analysis. Factors associated with CMvD with a *P* ≤ 0.20 were evaluated in multivariate logistic regression models. Before an introduction into the multivariate analysis, collinearity between the independent variables was evaluated using correlation analysis, and variables showing a correlation coefficient >0.5 were analyzed in separate multivariate models. As measurements from both eyes of the same participant are likely to be correlated, the standard statistical methods for parameter estimation lead to the underestimation of standard errors and to confidence intervals that are too narrow.^22^ Therefore, the clustered sandwich estimator technique was used in the logistic regression models, and the cluster of data for the study participant was considered as the primary sampling unit when estimating the coefficients and standard errors.^22^,^23^ Statistical analyses were performed using Stata version 13.1 (StataCorp, College Station, TX, USA) statistical software. A *P* ≤ 0.05 was considered statistically significant for the final analysis.

## Results

A total of 43 PXG eyes (36 patients) were enrolled for the study of which 4 eyes (three patients) were excluded because of poor quality OCTA scans (signal strength index < 35 and/or motion artifacts). The final analysis included 39 PXG eyes (33 patients) and 39 severity-matched POAG eyes (34 patients). The clinical features, VF parameters, peripapillary RNFL, and macular GCC thickness measurements of the included eyes are shown in Table 1. Global indices on VF, type of VF defect, RNFL thickness, and GCC thickness were comparable between the PXG and POAG eyes. Table 2 shows the peripapillary and parafoveal superficial retinal vessel density measurements and the parameters evaluated on the choroidal OCTA slabs in these eyes. Although most of the superficial peripapillary and macular vessel density measurements were lesser in PXG when compared with POAG eyes, none of the differences were statistically significant. Interexaminer agreement in detection of the CMvD was excellent (κ = 0.91, 95% confidence interval, 0.84–0.98). Intraclass correlation coefficients for the measurements of the choroidal OCTA slab were 0.92 (95% confidence interval, 0.85–0.98) for the disc area, 0.81 (0.70–0.92) for the PPA area, 0.82 (0.64–0.95) for the CMvD area, and 0.84 (0.68–0.93) for the CMvD angle. The prevalence of CMvD was significantly higher in the POAG eyes (79.5%) when compared with the PXG eyes (46.2%). The area of CMvD and its angular extent in the 18 PXG and 31 POAG eyes with CMvD were similar in both groups.

**Table 1:** Clinical Features, Visual Field Parameters, and OCT Measurements of the Participants in the PXG and POAG Groups

Variables	**PXG Group**	**POAG Group**	***P***
	**(39 Eyes, 33 Subjects)**	**(39 Eyes, 34 Subjects)**	
Age, y	66.3 ± 7.4	60.5 ± 13.6	0.04
Sex, male:female	21:12	23:11	0.73
Hypertension, n (%)	18 (54.6)	17 (50.0)	0.71
Diabetes mellitus, n (%)	13 (39.4)	12 (35.3)	0.73
Spherical equivalent, D	0.4 ± 1.5	−0.2 ± 2.0	0.2
Pseudophakia, n (%)	11 (28.2)	5 (12.8)	0.09
IOP at the scanning visit, mm Hg	19.1 ± 6.4	17.0 ± 4.1	0.09
No. of IOP lowering medications	1.87 ±1.2	1.62 ± 1.1	0.34
Type of IOP lowering medications			
Beta-blockers, n (%)	17 (43.6)	18 (46.2)	0.82
Prostaglandin analogues, n (%)	25 (64.1)	23 (59.0)	0.64
Alpha agonists, n (%)	18 (46.1)	9 (23.1)	0.03
Carbonic anhydrase inhibitors, n (%)	13 (33.3)	12 (30.8)	0.81
Disc hemorrhage, n (%)	7 (18.0)	8 (20.5)	0.77
Mean deviation, dB	−12.1 ± 8.5	−12.0 ± 8.3	0.96
Visual field index, %	68 ± 27	63 ± 27	0.36
Type of visual field defect, n (%)			
Nasal defect	4 (10.3)	4 (10.3)	
Parafoveal scotoma	2 (5.1)	2 (5.1)	1
Combined defect	33 (84.6)	33 (84.6)	
SSI, ONH scan of OCT	50 ± 6	49 ± 7	0.85
Peripapillary RNFL thickness, μm	71 ± 15	74 ± 12	0.29
Temporal sector RNFL thickness, μm	61 ± 12	64 ± 12	0.32
Superior sector RNFL thickness, μm	88 ± 23	93 ± 22	0.27
Nasal sector RNFL thickness, μm	61 ± 15	63 ± 12	0.63
Inferior sector RNFL thickness, μm	81 ± 21	78 ± 19	0.63
SSI, GCC scan of OCT	60 ± 7	62 ± 8	0.44
Average GCC thickness, μm	75 ± 11	75 ± 10	0.86
Superior GCC thickness, μm	77 ± 12	80 ± 10	0.26
Inferior GCC thickness, μm	72 ± 13	69 ± 12	0.18

All values represent mean ± standard deviation unless specified. SSI, signal strength index.

**Table 2:** OCT Angiography Measurements of the Participants in PXG and POAG Groups

OCTA Parameters	**PXG Group**	**POAG Group**	***P***
RPC slab measurements			
SSI, optic disc scan	49 ± 6	50 ± 6	0.41
Average peripapillary VD, %	49.7 ± 8.2	51.3 ± 6.9	0.35
Nasal sector VD, %	47.6 ± 8.9	50.1 ± 7.6	0.20
Inferonasal sector VD, %	48.6 ± 9.9	50.2 ± 11.1	0.50
Inferotemporal sector VD, %	47.2 ± 10.6	44.1 ± 11.6	0.22
Superotemporal sector VD, %	51.0 ± 13.4	54.6 ± 11.0	0.20
Superonasal sector VD, %	49.7 ± 9.1	52.4 ± 8.7	0.19
Temporal sector VD, %	53.4 ± 8.0	54.7 ± 6.4	0.43
Superficial macular measurements			
SSI, macular scan	58.9 ± 5.7	60.7 ± 6.2	0.19
Average parafoveal VD, %	44.8 ± 4.2	45.8 ± 3.7	0.33
Superior parafoveal VD, %	45.3 ± 4.6	45.6 ± 4.1	0.73
Inferior parafoveal VD, %	44.3 ± 4.7	44.8 ± 4.3	0.66
Temporal sector VD, %	44.9 ± 4.1	45.1 ± 4.1	0.87
Superior sector VD, %	45.6 ± 4.6	46.4 ± 4.0	0.43
Nasal sector VD, %	44.5 ± 4.7	45.2 ± 3.7	0.49
Inferior sector VD, %	44.0 ± 4.9	45.5 ± 4.7	0.21
Choroidal slab measurements			
Optic disc area, mm^2^	1.89 ± 0.45	2.06 ± 0.59	0.17
Parapapillary atrophy area, mm^2^	1.63 ± 0.51	1.57 ± 0.52	0.60
Presence of CMvD, n (%)	18 (46.2)	31 (79.5)	0.002
CMvD area, mm^2^	0.12 ± 0.13	0.11 ± 0.12	0.82
Angular extent of CMvD, degrees	49 ± 37	47 ± 34	0.83

All values represent mean ± standard deviation unless specified.

Table 3 shows the factors associated with the presence of CMvD in the entire cohort of open angle glaucoma eyes. On univariate analysis, female sex, lower IOP at scanning visit, absence of PXF, lower MD, lower visual field index (VFI), lower average RNFL thickness, and lower average GCC thickness were associated with higher odds of CMvD (at *P* ≤ 0.20). As MD, VFI, average RNFL, and GCC thickness (all being measures of glaucoma severity) were strongly correlated with each other (pairwise correlation coefficient, *r* > 0.60), separate multivariate models were built to evaluate the factors associated with CMvD. Table 3 shows two such multivariate models: one including VFI and the other including GCC thickness. CMvD was statistically significantly associated with PXF and the measures of glaucoma severity (coefficient of determination, *R*^2^ = 0.19). MD (odds ratio [OR] = 0.94; *P* = 0.05) and average RNFL thickness (OR = 0.95; *P* = 0.02) in the other two multivariate models (*R*^2^ = 0.19 in both models) also showed an association with CMvD.

**Table 3:** Factors Associated With the Presence of Choroidal Microvascular Dropout in Eyes With Open Angle Glaucoma

Factors	**Univariate Analysis**		**Multivariate Analysis 1**		**Multivariate Analysis 2**	
	**OR (95% CI)**	***P***	**OR (95% CI)**	***P***	**OR (95% CI)**	***P***
Age	0.97 (0.93–1.02)	0.30				
Male sex	0.46 (0.17–1.29)	0.14	0.32 (0.10–1.04)	0.06	0.41 (0.13–1.29)	0.13
Hypertension	0.62 (0.23–1.71)	0.36				
Diabetes mellitus	0.69 (0.26–1.85)	0.46				
IOP at scanning visit	0.94 (0.85–1.03)	0.18	0.93 (0.85–1.01)	0.11	0.94 (0.85–1.04)	0.20
PXF	0.22 (0.08–0.62)	0.004	0.24 (0.08–0.73)	0.01	0.21 (0.07–0.64)	0.006
Disc hemorrhage	0.87 (0.27–2.74)	0.80				
Disc area	1.19 (0.51–2.75)	0.69				
Area of PPA	0.91 (0.33–2.46)	0.84				
VF MD	0.96 (0.91–1.02)	0.19				
VF VFI	0.98 (0.96–1.00)	0.06	0.97 (0.95–0.99)	0.02		
Average RNFLT	0.97 (0.93–1.01)	0.20				
Average GCCT	0.95 (0.89–1.00)	0.07			0.94 (0.89–0.99)	0.01
Peripapillary VD	0.98 (0.92–1.05)	0.60				
Parafoveal VD	0.99 (0.88–1.13)	0.92				

CI, confidence interval; RNFLT, retinal nerve fiber layer thickness; GCCT, ganglion cell complex thickness.

The entire analysis was also repeated considering one eye from patients contributing both eyes to the primary analysis: 33 eyes of 33 PXG patients (mean MD = −11.4 ± 8.0 decibel [dB]) and 33 eyes of 33 POAG patients (mean MD = −11.6 ± 8.5 dB). One eye was chosen with the intention of matching the MD of both the groups to within 1 dB of MD. This matching was performed by a person masked to all details of the patients (including the CMvD details) except the type of glaucoma (PXG or POAG) and the VF MD values. The results were similar, with CMvD seen more frequently (*P* = 0.01) in the POAG eyes (26/33, 78.8%) when compared with the PXG eyes (16/33, 48.5%).

The analysis was also repeated in PXG and POAG groups separately. The factors associated with CMvD in the POAG eyes in the multivariate model (*R*^2^ = 0.20) were IOP at scanning visit (OR = 0.80; *P* = 0.05) and average RNFL thickness (OR = 0.93; *P* = 0.02). The factors associated with CMvD in the PXG eyes in the multivariate model (*R*^2^ = 0.19) were age (OR = 0.89; *P* = 0.03) and average GCC thickness (OR = 0.93; *P* = 0.07).

## Discussion

The current study found that the prevalence of CMvD was significantly lower in the PXG eyes when compared with the POAG eyes. To the best of our knowledge, this is the first report on the prevalence of CMvD in PXG eyes.

Previous studies have evaluated the superficial retinal vessel densities in the peripapillary region in PXG and have found them to be lower than the vessel densities in severity-matched POAG eyes. Suwan et al.^15^ compared the peripapillary vessel densities in 43 PXG (mean MD = −7.4 dB) and 31 POAG eyes (mean MD = −9.3 dB) using custom software and found that the vessel densities were significantly less in the PXG eyes. The mean annular perfused capillary density was 30.7% in the PXG group versus 36.2% in POAG group (*P* < 0.001). Similarly, all of the peripapillary sectors had significantly lower mean vessel densities in the PXG group (28.8%–34.8%) when compared with the POAG group (34.2%–39.2%).^15^ One of the limitations of this study was a large difference in the mean age between the two groups (71.2 years in PXG vs. 60.7 years in POAG group, *P* < 0.001). Another study by Park et al.^16^ also compared the peripapillary vessel densities of the radial peripapillary capillary slab in 39 PXG (mean MD = −6.8 dB) and 39 POAG eyes (mean MD = −6.7 dB) using a swept-source OCTA device and custom software. These groups were matched not only for MD on VF but also for age, and the peripapillary vessel densities and RNFL thicknesses were evaluated in six sectors of the Garway-Heath map. They found that, although the mean vessel densities were lower in all of the sectors of the PXG group when compared with the POAG group, this difference was statistically significant only for the average (24.2% vs. 26.4%, *P* = 0.048), nasal (23.3% vs. 26.3%, *P* = 0.02) and infero-nasal (18.5% vs. 21.9%, *P* = 0.043) sectors.^16^ They also found that, although the groups were matched for glaucoma severity based on VFs, the nasal RNFL was thinner in the PXG eyes when compared with the POAG eyes. Therefore, the reduction in vessel density may have been secondary to the corresponding RNFL thinning—an association that was established earlier.^24^ The strength of the present study, which found no difference in the vessel densities of the radial peripapillary capillary between the groups, was that the PXG and POAG groups had no significant difference in VF parameters or RNFL thickness. The other possible explanation for difference in results between the current study and those done previously is that we included large vessels as well as microvasculature in our measurement of vessel density. One may question why large vessels were included in our analyses. The reason for this was that several previous studies on PXF syndrome have shown it to be associated with systemic vasculopathies involving large vessels (cardiovascular and cerebrovascular disease) as well as ocular vasculopathies of large vessels (retinal vein occlusions and reduced retrobulbar circulation).^2^,^3^ However, the current OCTA technology may not be sensitive enough to appreciate these changes in the large vessel flow and therefore no significant difference was seen between PXG and POAG in the present study. Recently, OCTA software has become available that allows the removal of large vessels while calculating vessel densities, and this has been shown to be more sensitive at identifying progression when compared with the analysis of all vessel (large vessels and capillaries) information.^25^

The current study also evaluated the parafoveal vessel densities and found these to be lower in the PXG eyes when compared with the POAG eyes; the differences again were not statistically significant. However, the current study was not specifically designed to evaluate the difference in superficial retinal vessel densities between the PXG and POAG eyes and therefore may have been underpowered for the same.

Based on these prior reports of lower superficial vessel densities in PXG eyes when compared with POAG eyes, the current study aimed at evaluating the differences in the deep choroidal vasculature between these groups. CMvD is a novel sign described on OCTA, which is characterized by the complete loss of choriocapillaris in the PPA. A multivariate analysis of POAG eyes has shown that CMvD was significantly associated with a lower peripapillary superficial vessel density and, therefore, we hypothesized a higher prevalence of CMvD in PXG.^12^ Contrary to our expectation, the prevalence of CMvD was found to be significantly lower in the PXG eyes when compared with the POAG eyes. A study that compared the prevalence of CMvD in primary angle closure glaucoma and POAG found that it was less frequently seen in primary angle closure glaucoma.^14^ Also, CMvD was more frequently seen in POAG eyes with lower baseline (pretreatment) IOP.^14^ CMvD was associated with lower IOP at the scanning visit in univariate analysis in the entire group of glaucoma patients and in multivariate analysis in the POAG group in the present study as well. Hence, we speculate that CMvD is a focal ischemic process that is less frequent in eyes developing glaucomatous damage at high IOP, which explains our finding in PXG.

Measures of glaucoma severity were also associated with CMvD in the current study, with CMvD being more prevalent in eyes with greater severity of structural and functional glaucomatous damage. This is consistent with the results of previous studies in which the prevalence of CMvD was found to be higher in eyes with more severe glaucomatous damage.^12^,^14^,^19^,^26^

The current study also found CMvD to be more common in women than men. This is a novel association that has not been previously reported. The sex distribution was similar in the PXG and POAG cohorts (as shown in Table 1), and the severity of glaucoma was also similar (*P* = 0.16) in men (MD = −13.0 ± 8.9 dB) and women (MD = −10.2 ± 7.1 dB). The association between sex and CMvD was therefore not confounded by glaucoma severity and type of glaucoma. One of the possible reasons for this association could be that the baseline IOP (which was available in 17 PXG and 37 POAG eyes) was significantly lesser (*P* = 0.03) in women (18.9 ± 4.1 mm Hg) when compared with men (25.7 ± 12.3 mm Hg). This again mirrors the study that reported a higher prevalence of CMvD in POAG eyes with lower baseline IOP.^14^ Future studies should validate this result.

The current study has a few limitations. The visualization of choroidal vasculature with the current OCTA technology is obfuscated by projection artifacts.^4^ Projection artifacts are signals from the superficial retinal vessels projecting onto the choroidal slab. The presence of projection artifacts could have caused an underdetection of CMvD, and this should be considered while interpreting the results of studies evaluating CMvD. The other minor limitation was that the PXG patients were slightly older than the POAG patients. The association between CMvD and the absence of PXF persisted in the analysis that accounted for the difference in age between the groups. Another drawback was that the study participants were recruited from patients visiting a tertiary care eye center. Hence, this may induce a selection bias, and the results of the study may not be extrapolated to the general population. Lastly, the effect of blood pressure, systemic antihypertensive medications, and ocular hypotensive drops on CMvD parameters was not assessed. However, a history of systemic hypertension was recorded, and this was similar between the PXG and POAG groups. Future studies evaluating the effect of blood pressure and IOP-lowering drugs on CMvD are warranted.

In conclusion, the prevalence of CMvD was lower in the PXG eyes when compared with the POAG eyes. This may suggest that the mechanism of optic nerve damage in these disease entities is different.
